# Leukocyte ADAM17 Regulates Acute Pulmonary Inflammation

**DOI:** 10.1371/journal.pone.0019938

**Published:** 2011-05-16

**Authors:** Patrick G. Arndt, Brian Strahan, Yue Wang, Chunmei Long, Keisuke Horiuchi, Bruce Walcheck

**Affiliations:** 1 Division of Pulmonary, Allergy and Critical Care Medicine, Department of Internal Medicine, University of Minnesota, St. Paul, Minnesota, United States of America; 2 Veterinary and Biomedical Sciences, University of Minnesota, St. Paul, Minnesota, United States of America; 3 Department of Orthopedic Surgery, School of Medicine, Keio University, Shinjuku-ku, Tokyo, Japan; University Hospital Freiburg, Germany

## Abstract

The transmembrane protease ADAM17 regulates the release and density of various leukocyte cell surface proteins that modulate inflammation, including L-selectin, TNF-α, and IL-6R. At this time, its *in vivo* substrates and role in pulmonary inflammation have not been directly examined. Using conditional ADAM17 knock-out mice, we investigated leukocyte ADAM17 in acute lung inflammation. Alveolar TNF-α levels were significantly reduced (>95%) in ADAM17-null mice following LPS administration, as was the shedding of L-selectin, a neutrophil-expressed adhesion molecule. Alveolar IL-6R levels, however, were reduced by only ≈25% in ADAM17-null mice, indicating that ADAM17 is not its primary sheddase in our model. Neutrophil infiltration into the alveolar compartment is a key event in the pathophysiology of acute airway inflammation. Following LPS inhalation, alveolar neutrophil levels and lung inflammation in ADAM17-null mice were overall reduced when compared to control mice. Interestingly, however, neutrophil recruitment to the alveolar compartment occurred earlier in ADAM17-null mice after exposure to LPS. This decrease in alveolar neutrophil recruitment in ADAM17-null mice was accompanied by significantly diminished alveolar levels of the neutrophil-tropic chemokines CXCL1 and CXCL5. Altogether, our study suggests that leukocyte ADAM17 promotes inflammation in the lung, and thus this sheddase may be a potential target in the design of pharmacologic therapies for acute lung injury.

## Introduction

Acute lung injury (ALI), along with its more severe form acute respiratory distress syndrome (ARDS), affects ≈200,000 persons annually in the United States with mortality rates still unexpectedly high [1]–[3]. Various events can incite ARDS, and the release of pleiotropic inflammatory mediators such as TNF-α plays a key role in the lung inflammation that occurs [4], [5]. TNF-α release activates leukocytes, endothelial cells, and parenchymal cells in the lung, and induces the production of various neutrophil chemoattractants [6]–[8]. Neutrophil infiltration into the alveolar airspace is a critical event in the pathophysiology of airway inflammation. These cells release, in part, various proteases and reactive oxygen species that facilitate progressive lung injury [9]–[15]. Accordingly, the identification of mechanisms that regulate pulmonary inflammation, and specifically the recruitment of neutrophils and the release of TNF-α, is critical for determining therapeutic targets to lessen lung injury.

During the inflammatory response, various cell surface proteins undergo ectodomain shedding, typically at a juxta-membrane site that leads to the release of a soluble extracellular domain fragment. A number of leukocyte determinants that undergo this regulated proteolytic process have an important role in modulating inflammation [16]. A disintegrin and metalloproteinase-17 (ADAM17), originally referred to as TNF-α converting enzyme (TACE) [17], [18], plays a broad role in mediating ectodomain shedding [19]. Hence, we hypothesized that ADAM17 may have an important regulatory function in pulmonary inflammation. However, examining the role of ADAM17 *in vivo* is challenging, as homozygous deletion of the *Adam17* gene results in perinatal lethality [20], [21]. To overcome this limitation, we have generated conditional ADAM17-null mice with an ADAM17 deficiency in all leukocytes [22]. These animals are viable and we show here that a deficiency of leukocyte-expressed ADAM17 markedly alters neutrophil infiltration into the lung with an overall diminution in their recruitment to the alveolar compartment during acute lung inflammation. We also address the relevance of L-selectin, IL-6R, and TNF-α as *in vivo* substrates of leukocyte ADAM17 in the lung.

## Results

### ADAM17 regulates L-selectin, TNF-α and IL-6R levels in the lung after LPS exposure

**Figure 1:**
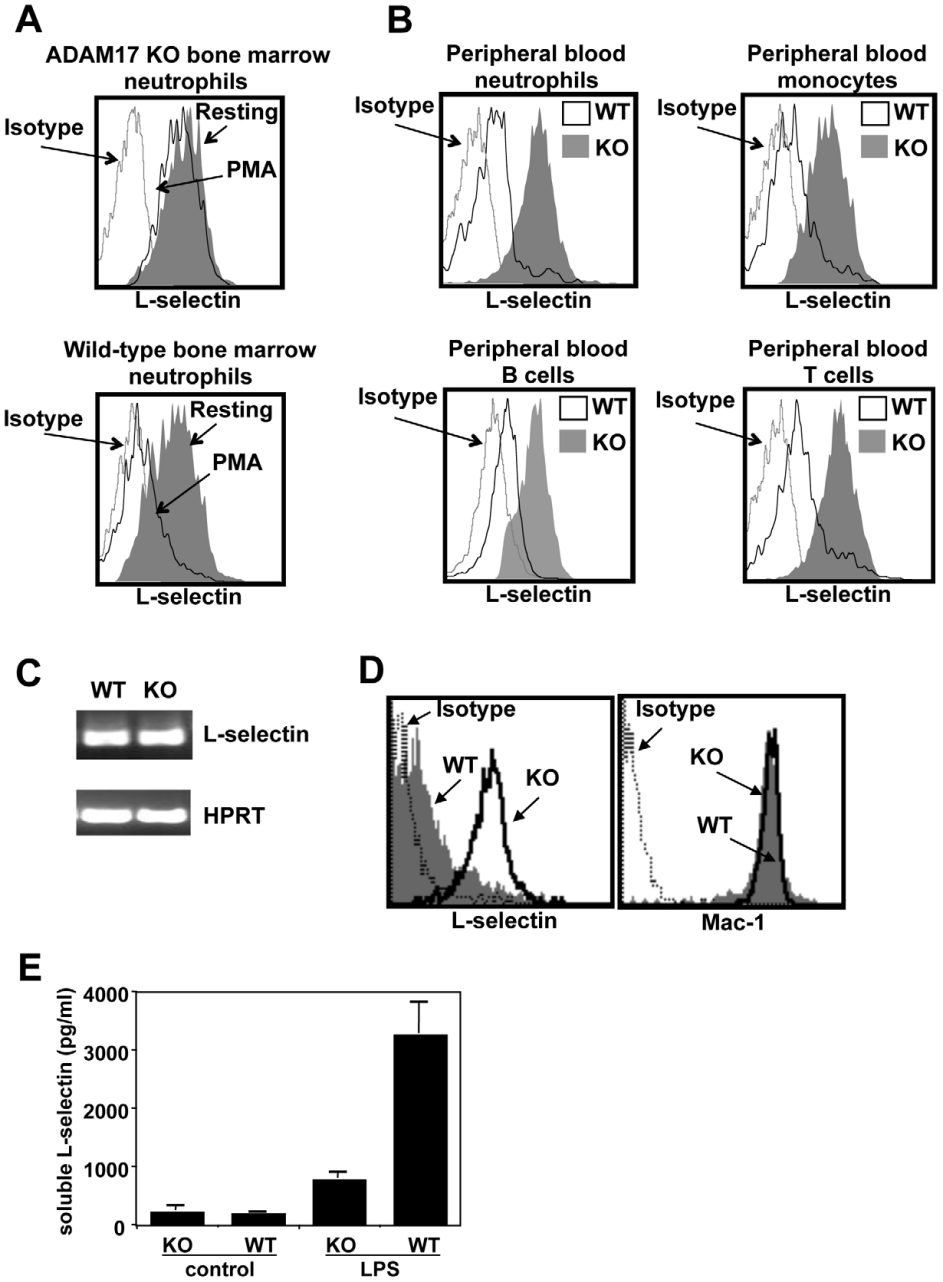
ADAM17 regulates alveolar neutrophil shedding of L-selectin. **A:** Bone marrow-derived leukocytes from ADAM17-null (KO) and control mice (wild-type) were treated with PMA or without (resting) for 30 minutes, as indicated, and then double-stained for surface expression of L-selectin and the neutrophil marker Ly-6G. **B:** Peripheral blood leukocytes from ADAM17-null (KO) and control mice (WT) were activated with PMA for 30 minutes and relative surface L-selectin expression levels were compared on neutrophils (Ly-6G^high^ CD11b^high^), monocytes (Ly-6G^−^ CD11b^high^), B cells (B220^+^), and T cells (CD3^+^), as indicated in the overlay plots. **C:** Detection of mouse L-selectin mRNA levels by semiquantitative RT-PCR was performed as described in the Materials and Methods. RNA was isolated from bone marrow neutrophils harvested from ADAM17-null and control mice. PCR amplification was performed for L-selectin and hypoxanthine phosphoribosyltransferase (HPRT), and the PCR products generated were 280 bp and 320 bp, respectively. Data are representative of three experiments using bone marrow neutrophils from separate mice. **D:** BAL leukocytes from ADAM17-null (KO) and control (WT) mice 2 hours after LPS inhalation were triple-stained for surface expression of Ly-6G, F4/80, and L-selectin or Mac-1. Leukocytes with the phenotypic profile Ly-6G^high^ F4/80^−^ were considered to be neutrophils. For panels A, B, and D, relative staining levels of 10,000 labeled cells were determined by flow cytometry. For all histogram plots, isotype-matched negative control antibody staining is indicated (Isotype). The y axis = cell number and the x axis = Log 10 fluorescence. Data are representative of at least 5 mice in each group. **E:** BAL fluid levels of soluble L-selectin from ADAM17-null (KO) and control mice (WT) either exposed to LPS for 8 hours or not (control). Results are expressed as mean ± SD of 4 mice in each group at each time point.

To examine if leukocyte ADAM17 can regulate pulmonary inflammation, we generated ADAM17-null mice [*Adam17^flox^* ^/ΔZn^/*Vav-Cre*] with an ADAM17 deficiency in leukocytes only. Similar to radiation chimeric mice reconstituted with ADAM17-deficient leukocytes that we have previously described [23], [24], our ADAM17-null mice were viable [22]. Cleavage of the well described ADAM17 substrate L-selectin was greatly impaired in neutrophils, monocytes, and lymphocytes from ADAM17-null mice following their overt activation *in vitro* when compared with the same leukocyte subsets from control mice (Fig. 1A, B). Despite ADAM17-deficient leukocytes expressing higher surface levels of L-selectin than control leukocytes, their levels of L-selectin mRNA were equivalent (Fig. 1C), indicating differential L-selectin shedding and not gene expression as a mechanism for their increased cell surface L-selectin levels. Neutrophil migration from the circulation into the underlying tissue at sites of inflammation results in L-selectin shedding [25]. We observed that alveolar neutrophils from ADAM17-null mice after LPS inhalation expressed significantly higher levels of surface L-selectin than alveolar neutrophils from control mice (Fig. 1D). Surface L-selectin levels were 5.4±2.5-fold higher on alveolar neutrophils from ADAM17-null mice than from control mice after LPS challenge (mean ± SD, n = 7 mice in each group). In contrast, the non-cleavable, cell surface adhesion molecule Mac-1 (CD11b/CD18) was expressed at equivalent levels by alveolar neutrophils from ADAM17-null and control mice following LPS instillation (Fig. 1D), demonstrating that ADAM17 deficiency did not cause a global up-regulation in the expression of cell surface molecules. Soluble L-selectin levels were also significantly reduced in the bronchoalveolar lavage (BAL) fluid of ADAM17-null mice when compared to control mice (Fig. 1E).

**Figure 2:**
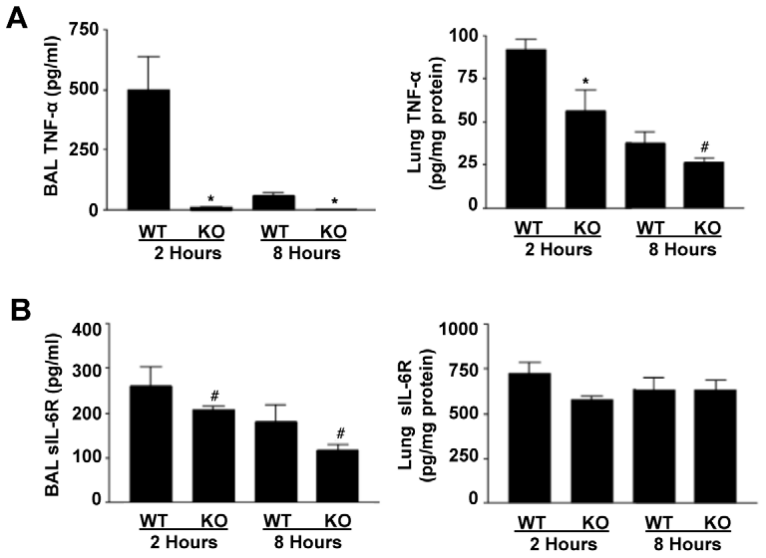
Decreased alveolar levels of TNF-α and IL-6R levels in ADAM17-null mice. Protein concentrations for TNF-α (**A**) and IL-6R (**B**) were measured by ELISA on BAL fluid (left panels) and lung homogenates (right panels) from ADAM17-null (KO) and control mice (WT) after LPS inhalation. Levels of TNF-α and soluble IL-6R in both groups of mice prior to LPS exposure were below the detection sensitivity of the ELISA (data not shown). * p<0.01 KO versus WT; # p<0.05 KO versus WT. Results are expressed as mean ± SD of at least 5 mice in each group at each time point. For the lung homogenates, data is expressed as pg/mg protein to normalize for the total amount of homogenate protein “loaded” in the ELISA well.

Neutrophils and macrophages are a primary source of soluble TNF-α upon the induction of inflammation [26], [27], and ADAM17 is a well described sheddase of TNF-α by these cells [17], [20], [21], [24]. We determined the levels of TNF-α in the BAL fluid of LPS exposed ADAM17-null and control mice, and found it to be greatly decreased (>95%) in ADAM17-null mice when compared to levels seen in control mice at time points that encompass its peak production after LPS challenge (Fig. 2A). Despite the near ablation of soluble TNF-α production in the alveolar compartment, the levels of TNF-α in the lung interstitial compartment were diminished by only ≈40% and 25% at 2 and 8 hours, respectively, following LPS inhalation in ADAM17-null mice (Fig. 2A), which may be due in part to ADAM17 expression by non-hematopoietic, lung parenchymal cells.

ADAM17 has been implicated as a key sheddase of the IL-6R as well [28]. We observed that soluble IL-6R levels were also significantly decreased in the alveolar compartment of ADAM17-null mice after LPS exposure (Fig. 2B), though this did not occur to nearly the same extent as TNF-α. For instance, at 2 hours after LPS exposure, soluble IL-6R levels in the airspace were ≈25% lower in ADAM17-null mice than in control mice (Fig. 2B). In the interstitial compartment, however, we did not observe a statistically significant difference in IL-6R levels in ADAM17-null mice after LPS exposure (Fig. 2B). It should be noted that our lung homogenizing buffer contained a detergent, and thus a portion of the TNF-α and IL-6R detected in the lung homogenates may represent membrane forms of these molecules. Taken together, the results above suggest that ADAM17 is the primary sheddase of L-selectin and TNF-α by alveolar leukocytes, but not of the IL-6R during acute pulmonary inflammation.

### Targeting leukocyte ADAM17 alters the time course and levels of pulmonary neutrophil recruitment

**Figure 3:**
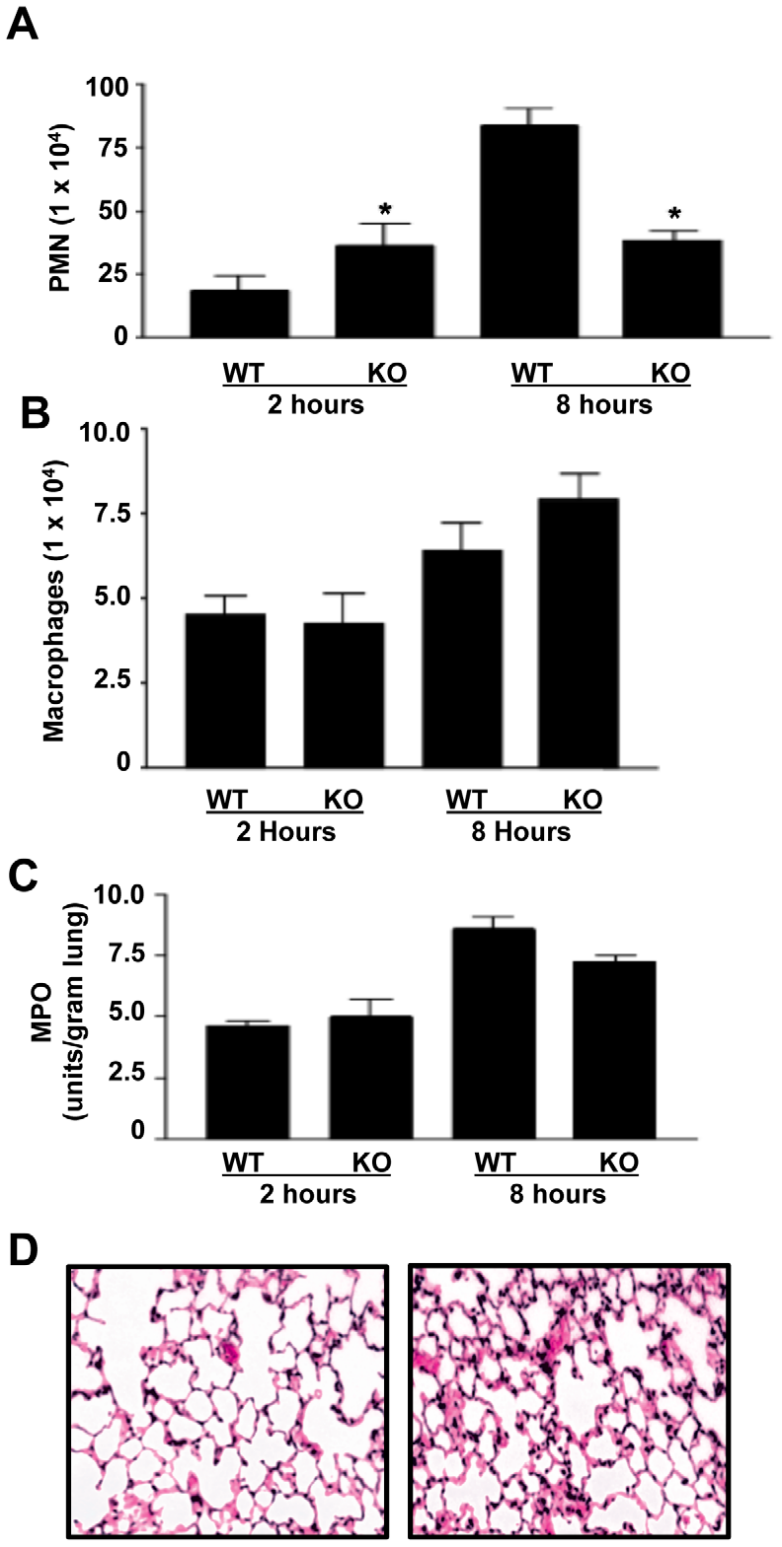
ADAM17 regulates alveolar neutrophil recruitment during acute pulmonary inflammation. **A:** ADAM17-null (KO) and control (WT) mice were exposed to aerosolized LPS. At the times indicated, mice were sacrificed and BAL was performed with total numbers of neutrophils determined. Results are expressed as mean ± SD of 5 mice in each group at each time point. * p<0.001 KO versus WT. **B:** Total numbers of BAL macrophages were determined in ADAM17-null (KO) and control (WT) mice at the indicated times following their exposure to aerosolized LPS. Results are expressed as mean ± SD of at least 3 mice in each group at each time point. **C:** MPO assays were performed on lung homogenates obtained from ADAM17-null and control mice exposed to LPS. MPO is expressed as activity per gram of lung tissue. **D:** Lungs from LPS exposed, ADAM17-null (left panel) and control mice (right panel) were isolated 8 hours after LPS exposure as described in the Materials and Methods. Lung sections (5 µm) were stained with hematoxylin and eosin. Note the increased number of cellular nuclei and thickened alveolar walls in the right panel. The stained tissue sections are shown at 100× magnification. Images are representative of 4 mice per group.

Neutrophil recruitment into the alveolar airspace is pivotal for inducing lung damage associated with an increase in mortality [11]. We compared the levels of neutrophil recruitment in the alveolar compartment of ADAM17-null and control mice. We have shown that ADAM17-null mice have similar leukocyte subset proportions in their peripheral blood and circulating neutrophil counts as control mice [22]–[24]. We observed, however, that 2 hours after exposure to aerosolized LPS, alveolar neutrophil counts were significantly higher in ADAM17-null mice (Fig. 3A). These results reveal that the significantly decreased levels of alveolar TNF-α and IL-6R observed in ADAM17-null mice at this time point, as discussed above, were not due to an attenuated influx of neutrophils into the alveolar compartment. Interestingly, we found that alveolar neutrophil counts were considerably lower in ADAM17-null mice at 8 hours after LPS inhalation (Fig. 3A), which represents the peak in alveolar neutrophil recruitment in our model [15], [29]. Alveolar macrophages, however, were not significantly different in ADAM17-null and control mice at either time point following LPS inhalation (Fig. 3B). In contrast to the alveolar air spaces, neutrophil levels in the interstitial compartment, as assessed by myeloperoxidase (MPO), did not differ between the two groups of mice at 2 or 8 hours after LPS challenge (Fig. 3C). These data indicate that the greatest effect of leukocyte ADAM17 was on regulating neutrophil movement to the alveolar compartment, and overall there was a marked decrease in acute lung inflammation, as revealed by histopathology (Fig. 3D).

### Alveolar levels of neutrophil chemoattractants are decreased after exposure to LPS in ADAM17-null mice

**Figure 4:**
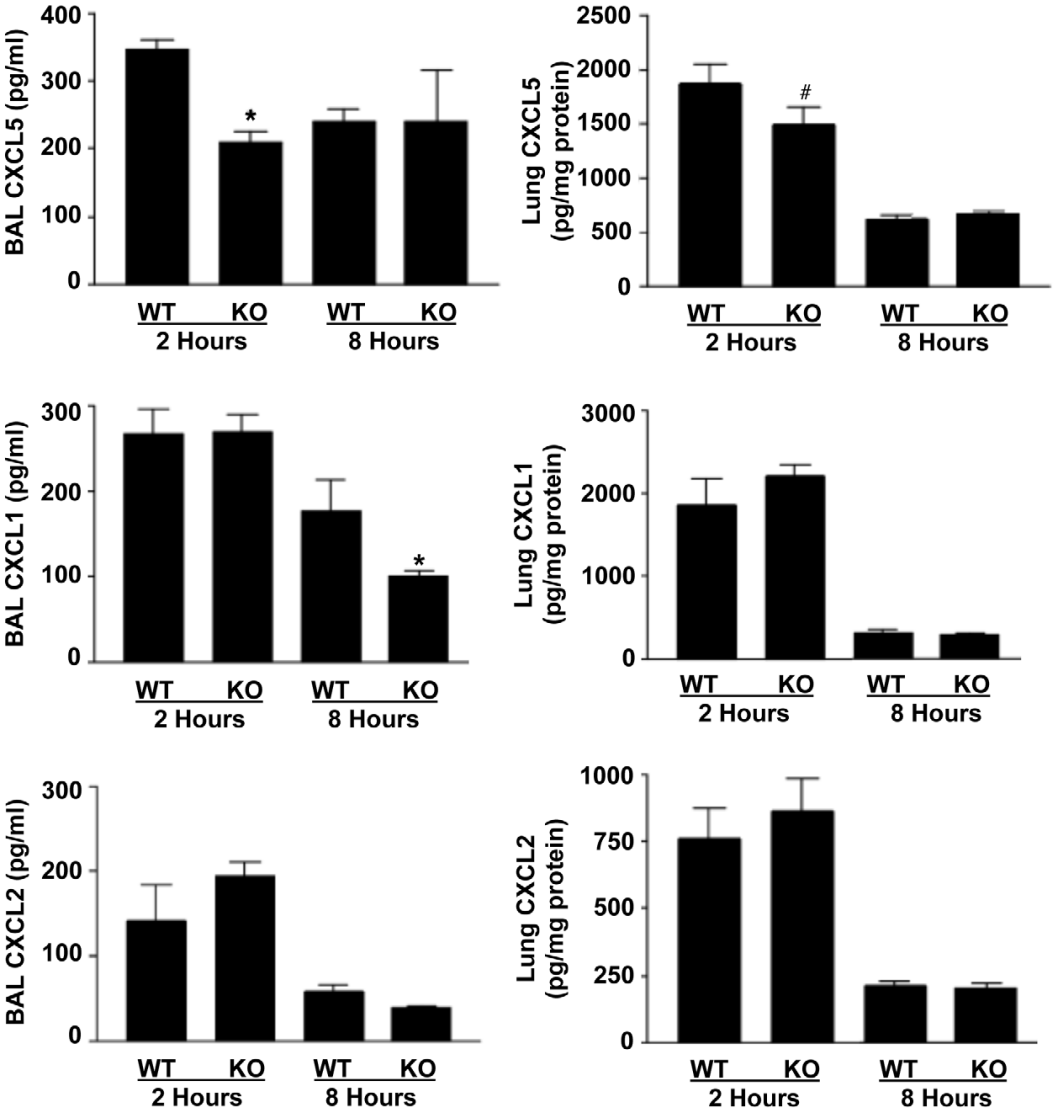
Leukocyte ADAM17 deficiency alters expression of the neutrophil chemoattractants CXCL1 (KC) and CXCL5 (LIX), but not CXCL2 (MIP-2), in the lung following LPS exposure. Protein concentrations for CXCL5, CXCL1, and CXCL2 were measured by ELISA on BAL fluid (left column) and lung homogenates (right column) from ADAM17-null (KO) and control (WT) mice after exposure to LPS for the indicated times. Levels of the chemokines in both groups of mice prior to LPS exposure were below the detection sensitivity of the ELISA (data not shown). * p<0.01 KO versus WT; # p<0.05 KO versus WT. Results are expressed as mean ± SD of at least 5 mice in each group at each time point. For the lung homogenates, data is expressed as pg/mg protein to normalize for the total amount of homogenate protein “loaded” in the ELISA well.

TNF-α has been reported to induce the expression of chemokines that promote pulmonary neutrophil recruitment [7], [8]. As levels of TNF-α were reduced in the alveolar and interstitial compartments of the lung after LPS exposure in ADAM17-null mice (Fig. 2A), this suggested that LPS-induced chemokine levels may also be diminished, thereby providing a mechanism for the decrease in alveolar neutrophil recruitment in ADAM17-null mice. To determine whether the production of chemokines important for neutrophil recruitment into the alveolar airspace is altered in ADAM17-null mice upon LPS inhalation, we examined the levels of CXCL1 (KC), CXCL2 (MIP-2), CXCL5 (LIX) in the BAL fluid. We observed that the alveolar concentrations of CXCL5 were significantly lower in ADAM17-null mice than control mice at 2 hours, but not 8 hours, after LPS instillation (Fig. 4). CXCL1 alveolar levels were also significantly lower in ADAM17-null mice, but only at 8 hours after LPS instillation (Fig. 4). In contrast to CXCL1 and CXCL5, alveolar levels of CXCL2 were not significantly different in the two groups of mice at 2 and 8 hours after LPS instillation (Fig. 4). Lung levels of CXCL5 were modestly lower at 2 hours, but not 8 hours, after LPS instillation in ADAM17-null mice, whereas lung levels of CXCL1 and CXCL2 were found not to differ between ADAM17-null and control mice (Fig. 4). The latter findings correspond with the similar level of neutrophils in the interstitial compartment after LPS exposure in the two groups of mice (Figure 3B).

## Discussion

In this study we report for the first time that ectodomain shedding by leukocyte ADAM17 participates in regulating neutrophil migration into the inflamed lung. Pulmonary neutrophil recruitment is a critical mechanism of pulmonary inflammatory disorders such as ARDS [9], [10], [12]–[15]. Excessive neutrophil recruitment into the alveolar compartment in particular has been associated with worsening lung damage and increased mortality [11]. We observed in ADAM17-null mice, whose leukocytes lack functional ADAM17, that neutrophil accumulation in the alveolar compartment, although occurring earlier, was overall significantly decreased compared to control mice following their exposure to LPS. This same pattern of rapid but attenuated neutrophil accumulation in the lung air spaces also occurred upon intratracheal injection of ADAM17-null mice with the Gram-positive bacterial constituent lipoteichoic acid (Arndt et al. unpublished results), a functional equivalent to LPS that instead induces neutrophil infiltration into the lung via TLR2. Neutrophil migration into the lung interstitial compartment, however, was not significantly decreased in ADAM17-null mice following LPS challenge, indicating that the targeting of leukocyte ADAM17 does not impair neutrophil infiltration into the interstitium from the pulmonary blood vessels, but does alter their advancement to the alveolar compartment.

ADAM17 expressed by neutrophils is a primary sheddase of L-selectin and TNF-α [23], [24]; however, its relevancy *in vivo* by neutrophils recruited to the inflamed lung has not previously been examined. We show that the conversion of membrane L-selectin and TNF-α to their soluble forms was greatly impaired in the inflamed lungs of ADAM17-null mice. In contrast to these substrates, the sheddase activity regulating the cleavage of IL-6R is in the early stages of being understood. The cleaved form of the IL-6R binds secreted IL-6 and enhances its pleiotropic activity through the activation of cells that lack expression of IL-6R via trans-signaling through the ubiquitously expressed glycoprotein 130 [30]. *In vitro* studies have implicated ADAM17 in IL-6R shedding [28], yet the biological relevance of ADAM17 in this process has not been directly investigated *in vivo*. Our results reveal that in the context of lung inflammation, ADAM17 participates in the shedding of IL-6R, but in contrast to TNF-α and L-selectin, ADAM17 is not the primary sheddase of leukocyte IL-6R. ADAM10 has also been reported to cleave the IL-6R [31], and thus it will be interesting to directly investigate its role in IL-6R shedding in a setting of acute lung inflammation.

It is well recognized that TNF-α induces an extensive array of downstream events that further promote inflammation, and thus the greatly diminished production of soluble TNF-α by ADAM17-deficient leukocytes likely contributed to the reduced levels of alveolar neutrophils as lung inflammation progressed after LPS exposure. For instance, TNF-α signaling has been directly shown to induce the production of neutrophil-tropic chemokines in the alveoli following LPS exposure [7], [8]. As CXCL1, CXCL2, and CXCL5 are major chemokines directing neutrophil recruitment into the murine lung [32]–[35], we examined their levels in the alveolar compartment of the lung in ADAM17-null and control mice. CXCL2 levels were found to be similar in the two groups of mice. However, CXCL5 and CXCL1 levels in ADAM17-null mice were significantly decreased at 2 hours and 8 hours, respectively, following LPS instillation. CXCL5 is primarily expressed by activated alveolar type II cells [36], and attenuated early production of soluble TNF-α by resident and recruited leukocytes in ADAM17-null mice may have delayed the activation of these cells and the initial production of CXCL5 in the airspace. CXCL1 is secreted by a variety of cells including neutrophils [34], [37]–[40], and the time frame of its reduction in alveolar levels in ADAM17-null mice corresponded with the marked reduction in alveolar neutrophil numbers as inflammation progressed following LPS exposure. In contrast to the alveolar spaces, only CXCL5 was decreased in the interstitial compartment of the lung in ADAM17-null mice. The greater reduction in neutrophil-tropic chemokines in the alveolar compartment of ADAM17-null mice implies a specific molecular process accounting for the lack of neutrophil transepithelial migration into the alveolar air spaces at the later time point in our study. Of additional interest is that alveolar neutrophil levels in ADAM17-null mice were initially enhanced upon inducing lung inflammation when compared to control mice. The reasons for this are not clear at this time, but may be the result of still other neutrophil chemoattractants or an enhanced ability of ADAM17-deficient neutrophils to respond to them.

In conclusion, our study demonstrates for the first time that leukocyte ADAM17 regulates acute lung inflammation by modulating intra-alveolar neutrophil levels and the shedding of IL-6R, L-selectin, and TNF-α. It is known that preventing TNF-α activity can increase host susceptibility to infection, and thus it will be important to determine the role of leukocyte ADAM17 in pulmonary defense against bacterial infection. Of interest is that we have recently reported that ADAM17-null mice demonstrate enhanced host resistance, including decreased hematogenous spread of bacterial to the lung, during *E. coli*-mediated abdominal sepsis [22].

## Materials and Methods

### Generation of conditional ADAM17 knock-out mice

Experimental procedures involving animals were approved by the Animal Care and Use Committee of the University of Minnesota and performed in accordance with the United States Public Health Service Policy on Humane Care and Use of Laboratory Animals and the Animal Welfare Act. *Tace^flox/flox^* mice [21], *Tace* ^ΔZn/+^ mice (Δ*Zn* represents a targeted deletion of exon 11 that encodes the catalytic active site of the ADAM17 metalloprotease domain, resulting in a lack of enzymatic activity) [20], and *Vav1-cre* transgenic mice [41], [42] have all been previously described. *Adam17* instead of *Tace* is the current gene symbol and will be used for our mouse colony nomenclature. *Vav1-cre* transgenic mice, which directs Cre-recombinase activity specifically in all hematopoietic cells [41], were crossed with *Adam17* ^ΔZn/+^ mice to produce *Adam17* ^ΔZn/+^/*Vav-Cre* mice. These mice, which are of mixed genetic background (129Sv, C57Bl/6), were then crossed with *Adam17^flox/flox^* mice to generate *Adam17^flox^* ^/ΔZn^/*Vav-Cre* and *Adam17^flox^* ^/ΔZn^ mice. This breeding scheme was used to avoid Cre activity in the germ line causing conversion of the floxed allele to a null allele and non-tissue specific *Adam17* gene deletion in the offspring. Littermate *Adam17^flox^* ^/ΔZn^ mice were used as controls since there is no described haploinsufficiency by *Adam17* [20]. Herein, the *Adam17^flox^* ^/ΔZn^/*Vav-Cre* and *Adam17^flox^* ^/ΔZn^ mice will be referred to as ADAM17-null and control mice, respectively. ADAM17-null and control mice were distinguished by PCR and functional screening. For the latter, we assessed shedding of the ADAM17 substrate L-selectin by peripheral blood neutrophils, monocytes, and lymphocytes, as previously described [22], [23]. ADAM17-null mice demonstrating essentially complete abrogation of L-selectin shedding by their leukocytes were used in this study.

### Aerosolized model of LPS-induced acute lung inflammation

Mice 8–12 weeks of age were used for our studies. LPS [*E. coli* O111:B4; 300 µg/ml in 0.9% saline (Sigma, St. Louis, MO)] was administered by aerosolization (20 minutes exposure period). Based on previous experiments, 2 and 8 hour time points were chosen as they represent the peaks in cytokine/chemokine levels and neutrophil influx, respectively [15], [29]. Animals were anesthetized with Avertin (240 mg/kg per mouse IP) 30 minutes prior to intratracheal inoculation. Mice were sacrificed by an overdose of sodium pentobarbital (100 mg/kg), blood was collected, BAL was performed, bone marrow isolated, and lungs were collected, snap frozen, and stored at −80°C until further analysis, as previously described [15], [43]. Total cell counts by hemocytometer and cell differentials of Wright stained cytocentrifuged samples were performed on BAL fluid. Endotoxin free glassware and plasticware were used in all experiments. MPO levels, as a quantification of the number of neutrophils recruited to the lung after exposure to LPS, were performed as we have previously described [15], [43]. For lung histology, mice did not undergo BAL and instead lungs were isolated, inflated, and fixed in 3.7% formalin overnight at room temperature as previously described [15].

### ELISA

ELISA for CXCL1, CXCL2, CXCL5, soluble IL-6R, L-selectin, and TNF-α were performed on BAL fluid and lung homogenate supernatants using commercially available kits (R&D Systems, Minneapolis, MN) following the manufacturer's protocols. Lungs were homogenized in PBS containing leupeptin (10 µg/ml), aprotinin (10 µg/ml), PMSF (300 mM), and 1% Triton X-100, centrifuged 10 minutes at 2,040*g* with supernatants collected and frozen at −80°C until assayed. For normalization of results for lung total protein, protein estimation on lung homogenates was performed using the Bradford assay kit following the manufacturer's instructions (Thermo Scientific, Rockford, IL).

### Cell labeling and flow cytometry

Leukocytes mice were stained with various mAbs for flow cytometry analysis, as we have previously described [23], [24].

### PCR

Detection of mouse L-selectin mRNA levels by semiquantitative RT-PCR was performed as described with modifications [44]. Bone marrow neutrophils were harvested from ADAM17-null and control mice and total cellular RNA was isolated from 5×10^6^ cells using a Qiagen RNeasy Mini Kit along with RNase-Free DNase to remove residual amounts of DNA, which were performed according to the manufacturer's instructions (Qiagen, Valencia, CA). Reverse transcription and PCR were performed sequentially using a Qiagen OneStep RT-PCR Kit and mouse L-selectin gene-specific primers, as per the manufacturer's instructions. PCR amplification was performed using the following primers (5′ to 3′): L-selectin, CATTCCTGTAGCCGTCATGG and AGGAGGAGCTGTTGGTCATG; hypoxanthine phosphoribosyltransferase (internal control), GTTGGATACAGGCCAGACTTTGTTG and GAAGGGTAGGCTGGCCTATAGGCT, which do not amplify genomic DNA. The PCR conditions consisted of 95°C for 15 min and 30 cycles of 94°C 30 sec; 57°C 30 sec; 72°C 40 sec, and a final 72°C for 10 min. Thirty cycles were determined to be below the plateau phase of amplification for all primers (data not shown), giving an accurate reflection of the relative starting levels of mRNA. PCR products were detected by 1.5% agarose gel electrophoresis.

### Statistical analysis

Statistical analysis was performed using Prism software (Prism 4; GraphPad, San Diego, CA) using ANOVA and student's t test where appropriate. A p value of <0.05 was considered significant.
